# Critical Factors Affecting the Success of Cloning, Expression, and Mass Production of Enzymes by Recombinant *E. coli*

**DOI:** 10.5402/2013/590587

**Published:** 2012-08-13

**Authors:** Md. Fakruddin, Reaz Mohammad Mazumdar, Khanjada Shahnewaj Bin Mannan, Abhijit Chowdhury, Md. Nur Hossain

**Affiliations:** ^1^Industrial Microbiology Laboratory, Institute of Food Science and Technology (IFST), Bangladesh Council of Scientific and Industrial Research (BCSIR), Dhaka 1205, Bangladesh; ^2^BCSIR Laboratories, Chittagong, Chittagong 4220, Bangladesh; ^3^Center for Food and Waterborne Diseases (CFWD), ICDDRB, Dhaka, Bangladesh

## Abstract

*E. coli* is the most frequently used host for production of enzymes and other proteins by recombinant DNA technology. *E. coli* is preferable for its relative simplicity, inexpensive and fast high-density cultivation, well-known genetics, and large number of compatible molecular tools available. Despite all these advantages, expression and production of recombinant enzymes are not always successful and often result in insoluble and nonfunctional proteins. There are many factors that affect the success of cloning, expression, and mass production of enzymes by recombinant *E. coli*. In this paper, these critical factors and approaches to overcome these obstacles are summarized focusing controlled expression of target protein/enzyme in an unmodified form at industrial level.

## 1. Introduction

In the past few years recombinant DNA technology has enabled scientists to produce a large number of diverse proteins, in microorganisms, that were previously unavailable, relatively expensive, or difficult to obtain in quantity [1]. While the expression of foreign genes has been reported in a variety of microorganisms and cell lines, most of this work utilizes *E. coli* for the cloning and expression of foreign genes [2]. Production of enzymes involves cloning of the appropriate gene into an expression vector under the control of an inducible promoter [3].

## 2. Enzyme Production in *E. coli*

The expression of recombinant proteins in cells in which they do not naturally occur is termed *heterologous protein production*. Bacterial expression systems are commonly used for production of heterologous gene products of both eukaryotic and prokaryotic origin [4]. The expression of heterologous proteins in *E. coli*, which is the bacterial system, is most widely and routinely used. A number of therapeutically important proteins are now produced as heterologous in *E. coli*. The first heterologous protein to be employed clinically was human insulin produced in *E. coli*, first approved in 1982, in UK, West Germany, The Netherland, and USA [5] (Table 1).

## 3. General Considerations of Selecting *E. coli* as Heterogeneous Protein Expression Host

*E. coli* is widely used as the host for heterogeneous protein expression for the following advantages: (1) ease of growth and manipulation using simple laboratory equipment; (2) availability of dozens of vectors and host strains that have been developed for maximizing expression; (3) a wealth of knowledge about the genetics and physiology of *E. coli*; (4) expression can often be achieved quite rapidly beginning with an eukaryotic cDNA clone, express the protein in *E. coli*, and purify in milligram quantities in less than 2 weeks; (5) suitable fermentation technology well established; (6) can generate potentially unlimited supplies of recombinant protein; (7) economically attractive [6].

## 4. Limitations Using *E. coli* as Heterogeneous Protein Expression Host

They are (1) inability of *E. coli* as a prokaryotic to carry out posttranslational modification which is typical for eukaryotic; (2) limited ability to carry out extensive disulfide bond formation; (3) some proteins are made in insoluble form, a consequence of protein misfolding, aggregation, and intracellular accumulation as inclusion bodies; (4) sometimes sufficient expression may not be observed due to protein degradation or insufficient translation (mRNA may remain in secondary structure and translation hampered); (5) codon sequence for a specific amino acid in Eukaryotic is different from Prokaryotic as *E. coli. *This phenomenon is known as “codon bias” which vastly hampers protein synthesis and gene expression in *E. coli *[6]. 

## 5. Factors Affecting Expression of Enzymes in *E. coli*

The expression of genes of enzymes in *E. coli* is influenced by a range of factors. These are discussed below.

### 5.1. Unique and Subtle Structural Features of the Gene Sequence

Unique DNA sequences are involved in different stages of expression of recombinant enzymes such as transcription and translation.

(a)* DNA Sequences Involved in Transcription.* Three different DNA sequences and one multicomponent protein are involved in transcription of genes. (1) *The promoter*: promoters normally consist of three regions called the −35 and the −10 box and the spacer region separating both boxes. Alignment of many promoters allows the deduction of a so-called consensus sequence. This sequence represents the optimal promoter sequence with a spacer region of 17 nucleotides. It should be mentioned that there is not a single promoter present on the *E. coli *chromosome identical to the consensus sequence. In most cases, there are one or two deviations in both the −35 and the −10 box [4]. (2) *The transcriptional terminator*: a transcriptional terminator is required to allow termination of transcription. Two classes of terminators have been described, factor-independent and factor-dependent terminators [7]. (3) *The regulatory sequence*: genes are either expressed constitutively or regulated. Two different classes of regulators have been described, transcriptional repressors and transcriptional activators. Repressors bind to operators located either within the promoter region or immediately downstream from it and, in most cases, prevent RNA polymerase promoter binding or act as a road block. To relieve repression, the repressor has to dissociate from its operator. In some cases, an inducer will be either synthesized by the cell or taken up from the environment which binds to the repressor causing dissociation from its operator [3]. (4) *The RNA polymerase*: the RNA polymerase consists of five different components termed *α*, *β*, *β*′, *ω*, and *σ*. While *α*_2_ *ββω* constitute the core enzyme, addition of *σ* conferring promoter specificity makes up the holoenzyme. The *σ* factor is responsible for the recognition of the promoter, and it follows that each *σ* factor recognizes a different promoter [8]. *E. coli *codes for six alternative factors where *σ* ^32^ is needed after a sudden temperature upshift and *σ* ^S^ replaces the housekeeping *σ* factor *σ* ^70^ during the stationary phase. So far, only *σ* ^70^ is used in the production of recombinant proteins such as enzymes [3].

*(b) DNA Sequences Involved in Translation*. It became clear that the wide range of efficiencies in translation of different mRNAs is predominantly due to the structure at the 5′ end of each mRNA species. The translation initiation region comprises four different sequences: (1) the Shine-Dalgarno sequence, (2) the start codon, (3) the spacer region between the Shine-Dalgarno sequence and the start codon, the optimal spacing has been determined to be 4 to 8 nucleotides, and (4) translational enhancers [3].

The secondary structure at the translation initiation region of the mRNA plays an important role in the efficiency of gene expression. It has been shown that occlusion of the Shine-Dalgarno sequence and/or the start codon by a stem-loop structure prevents accessibility to the 30S ribosomal subunit and inhibits translation [9]. The mutation of specific nucleotides up- or downstream from the Shine-Dalgarno sequence suppressed the formation of mRNA secondary structures and enhanced the translation efficiency [10, 11].

### 5.2. The “Strength” of the Transcriptional Promoter

For higher expression, the gene of enzymes should be placed under the control of a strong promoter. Many plasmid and bacteriophage vectors have been developed in which the cloned gene is situated immediately downstream from a strong transcriptional promoter [2]. Use of these vectors requires that the promoter should not be constitutive (i.e., always turned on) but, rather, be turned on at a specific stage in the growth of the transformed *E. coli* cells. This is often accomplished by the addition of a specific metabolite or by a shift in the temperature of the growth medium [12]. Regulation of promoter activity ensures that the expression of a foreign gene does not interfere with normal cellular gene functions and is not deleterious to the cell. Failure to regulate the expression of strong promoters often results in the loss of the plasmid carrying the strong promoter or the constitutive expression of the strong promoter which may be lethal to the cell [13].

The most widely used strong promoters are from the *E. coli* trp and lac operons, the tae promoter (an *in vitro* construct including elements from both the trp and lac promoters), and the leftward, or pL, promoter of bacteriophage lambda [4]. 

### 5.3. The Stability of the Vector in *E. coli* Cells

After a foreign gene has been cloned into an expression vector, the vector is introduced into competent *E. coli* cells that become a source of the foreign protein. However, plasmids are not always stable, especially in cells grown for many generations in large-scale cultures [14] so that when a process is scaled up it is important that vector stability be addressed. Since a plasmid-free strain has a faster-specific growth rate than a plasmid-containing strain, as a result of the metabolic energy which is expended for plasmid maintenance, the plasmid-free strain will eventually outcompete the plasmid-containing strain [15].

#### 5.3.1. Reasons of Instability

(1) Plasmid stability is influenced by the vector and host genotypes; the same plasmid in different hosts exhibits different degrees of stability and vice versa [16]. (2) The origin and size of foreign DNA have been observed to affect the plasmid stability [16]. (3) Plasmid loss first occurs at the level of the individual cell as a result of defective segregation at cell division, and then at the population level [15]. (4) Instability is due to increase in metabolic energy required for plasmid maintenance and function [17]. (5) Plasmid stability is also a function of physiological parameters that affect the growth rate of the host cell, which include pH, temperature, aeration rate, medium components, and heterologous protein accumulation [16].

#### 5.3.2. Solutions to the Problem of Instability

(1) The most common method of ensuring that a recombinant plasmid is not lost during the growth of the microorganism is the inclusion of antibiotics which are selected for the presence of plasmids carrying the appropriate antibiotic resistance genes. However, scale-up of this approach may not be economically feasible due to the cost of the added antibiotics placed on the cell [14]. (2) An analogous strategy involves the use of runaway-replication plasmid vectors where plasmid copy number is relatively low at lower temperatures and is increased when the temperature is raised. The lower plasmid copy number during much of the cell growth cycle reduces the metabolic load on the cell and ensures plasmid stability. At the same time the higher plasmid copy number for a portion of the growth cycle results in high levels of expression of the cloned foreign gene [18].

### 5.4. The Number of Copies of the Gene

Since the target gene is often incorporated into a plasmid vector system, gene dosage is dependent on plasmid copy number. As can be expected, an increase in copy number results in concomitantly higher recombinant protein productivity, but not indefinitely. Plasmid copy number is affected by plasmid and host genetics and also by cultivation conditions such as growth rates, media, and temperature [19]. 

### 5.5. Codons Utilized in Foreign Gene Compared to the Normal Pattern of Codon Usage in *E. coli*

Since the 20 amino acids are encoded by 61 different trinucleotide codons, several trinucleotide codons can encode the information for the insertion of the same amino acid into protein. Organisms show marked differences in codon preference. In fact, it appears that the frequency of codon usage in an organism is a direct reflection of the pool of cognate tRNAs [20]. Highly expressed genes use codons for which there is a large pool of cognate tRNAs while regulatory genes often use codons for which there is only a very small pool of cognate tRNAs. Accordingly, expression of a foreign gene may be limited by the availability of a particular aminoacyl tRNA [21]. 

The codon usage by the different species can be quite different. As an example, codon usage for arginine of four different species is presented in the following Table 2.

Overexpression of genes with high contents of rare codons may result in defective synthesis of the corresponding enzyme. Besides the amount, the location of rare codons within the coding region can significantly influence the translation level. Rare codons close to the initiator may stall the ribosome and prevent the entry of new incoming ribosomes [22].

#### 5.5.1. Solutions to the Problem of Codon Usage

There are two experimental solutions to this problem: (1) increase in the amount of the appropriate cognate tRNA, (2) alteration of these codons to frequently used ones by sequence-specific mutagenesis [22]. 

### 5.6. The Stability and Efficiency of mRNA

mRNA of recombinant genes tends to accumulate in the cell; however, *E. coli *mRNAs are rather unstable. Some features of mRNA affect its stability. These include (1) the Shine-Dalgarno (S-D) sequence at the 5′ end of the mRNA that is thought to help position the mRNA on the ribosome, (2) the distance between the S-D sequence and the initiation codon, and (3) the secondary and tertiary structure of the mRNA [7]. 

#### 5.6.1. Solutions

(1) It was reported recently that the addition of a short-specific DNA sequence (approximately 89 base pairs) to the distal end of cloned genes may stabilize the mRNA transcribed from that gene, thereby increasing gene expression. This “retroregulator” sequence probably becomes incorporated at the 3′ end of the mRNA, protecting it from exonuclease digestion [23]. (2) It has been shown that stable secondary structures engineered into the 5′ untranslated region and 3′ rho-independent terminator of the mRNA can aid in mRNA stability and prevent degradation by exonucleases. In particular, a hairpin at the 5′ end without any 5′ single-stranded nucleotide overhangs has conferred mRNAs with considerable resistance to exonuclease activity in the cytoplasm [24].

### 5.7. The Location of the Cloned Protein within the *E. coli* Cell

While *E. coli* proteins are synthesized in the cytoplasm, it is possible to direct a cloned gene product to the cytoplasm, the inner or outer membrane, or the periplasmic space [25]. Secretion of a cloned gene product to the periplasmic space often allows for higher levels of expression of the foreign protein that might be degraded by proteases in the cytoplasm [26]. *E. coli* is capable of recognizing and correctly processing signal sequences so that secretion of enzymes into the *E. coli* periplasmic space is possible [27].

#### 5.7.1. There are Four Reasons to Translocate Recombinant Proteins into the Periplasm

(1) the oxidizing environment facilitates the formation of disulfide bonds, (2) it contains only 4% of the total cell protein (~100 different proteins), (3) there is less protein degradation, and (4) easy purification by osmotic shock [3].

#### 5.7.2. Disadvantage of Periplasmic Expression

While it is technically feasible to direct the protein products of foreign genes to the inner or outer membrane, high levels of a foreign protein in the membrane may interfere with normal cellular functions and be lethal to the cell [28]. 

#### 5.7.3. Solution

Expression vectors have recently been constructed which place the genes for foreign proteins, not normally secreted, behind a DNA fragment encoding a signal sequence. This results in the foreign protein being efficiently secreted (in large amounts) to the periplasmic space with no evidence for accumulation of the unprocessed form in the cytoplasm [29]. 

### 5.8. The Stability of the Cloned Enzyme in *E. coli*

Secretion of a cloned gene product to the periplasmic space often allows for higher levels of expression of the foreign protein that might be degraded by proteases in the cytoplasm [26]. The large-scale production of eukaryotic proteins in *E. coli* is often limited by the instability of these polypeptides within the bacterial host [30]. 

Protease susceptibility can be affected by the N- and C-terminal sequences of the recombinant protein. The presence of Arg, Leu, Lys, Phe, Trp, or Tyr at the N-terminus targets proteins for more rapid degradation (N-end rule). Nonpolar amino acids at the C-terminus can lead to rapid degradation; however, proteins with last five amino acids polar or charged fail to be degraded [31]. 

Other factors in protease susceptibility include (1) the presence of damaged or excess protein products caused by formation of incomplete polypeptides, (2) excessive synthesis of subunits from multimeric complexes, (3) post-translational damage, or genetic engineering of the target protein, and (4) culture growth parameters such as nutrient composition of media, growth temperature, and pH [32].

#### 5.8.1. Solving the Problem

(1) A common strategy which has been used to overcome this problem is to fuse the gene for the eukaryotic protein to a portion of a bacterial gene [33]. (2) An alternate approach to stabilizing a cloned protein is to clone multiple copies of the gene in tandem onto the same plasmid [34]. 

### 5.9. Inclusion Bodies and How to Prevent Their Formation

Rapid production of recombinant proteins can lead to the formation of insoluble aggregates designated as inclusion bodies [35]. These are large, spherical particles which are clearly separated from the cytoplasm and result from the failure of the quality control system to repair or remove misfolded or unfolded protein [36]. In this instance it may be advantageous to clone the gene into a secretion vector so that the cloned protein does not accumulate in the cytoplasm [37].

#### 5.9.1. Solutions

Strategies to prevent the formation of inclusion bodies are aimed to slow down the production of recombinant proteins and include (1) low-copy number vectors, (2) weak promoters, (3) low temperature, (4) coexpression of molecular chaperones, (5) use of a solubilizing partner, and (6) fermentation at extreme pH values [3]. (7) A common strategy which has been used to overcome this problem is to fuse the gene for the eukaryotic protein to a portion of a bacterial gene [33]. 

*Advantages of Expression or Heterologous Proteins as Fusion Proteins or with Protein Tag.* Many vectors are available which allow expression of heterologous proteins which are fused at their N- or C-terminal partners are often termed as protein tag [38]. For example, Histidine (His) tag is a fusion protein. Such fusion partners offer several potential advantages. *Improved expression*: fusion of the N terminals of a heterologous protein to the C-terminus of a highly expressed fusion partner often allows high level of expression of the fusion protein [39]. *Improved solubility*: fusion of N terminus of heterologous protein to the C-terminus of a soluble fusion partner often improves solubility of a protein [40]. *Improved detection*: fusion of a protein at either terminus to a short peptide or a polypeptide which is recognized by an antibody or binding protein allows western blot analysis of a protein during expression and purification [41]. *Improved purification*: it is a widely used phenomenon. Simple purification schemes have been described for proteins fused at either end to tags which bind affinity resins. Available tags include His_6_ (six tandem Histidine residues), which bind to Ni-NTA (nitrilotriacetate chelated with Ni^2+^ ions); GST (glutathione-S-transferase, which bind to glutathione-sepharose). These tags bind to their specific resins and separated easily. There is no effect of tags on protein and the excised easily [42]. 

### 5.10. Correct and Efficient Protein Folding

During or following translation, the polypeptide must fold so as to adopt its functionally active conformation [43]. Since many denatured proteins can be refolded *in vitro*, it appears that the information for correct folding is contained in the primary polypeptide structure [44]. However, folding comprises rate-limiting steps during which some molecules may aggregate, particularly at high rates of synthesis and at higher temperatures. In contrast to intracellular proteins, naturally secreted proteins encounter an abnormal environment in the cytoplasm; disulphide bond formation is not favoured and glycosylation cannot occur [45].

#### 5.10.1. Solutions

(1) Coexpress additional chaperones to aid in protein folding. This can cause a reduction in the expression of the enzyme, but it promotes solubility. There is evidence that certain heat shock proteins act as molecular chaperones in preventing the formation and accumulation of unfolded aggregates, while accelerating the folding reactions. (2) For disulfide bond formation, coexpress thioredoxin (or use as a fusion partner) or use strains deficient in thioredoxin reductase. An alternative to consider is targeting the protein to the periplasm where disulfide-bond formation can occur (most *E. coli *proteins having disulfide bonds are located in the periplasm) [46].

### 5.11. Cell Growth Characteristics

Cell growth characteristics have marked influence on the expression of recombinant enzymes. Some of the manipulations of culture media are as follows. (a) *Decrease culture growth temperature*: advantages of decreased growth temperature are the following. (1) Growth at 37°C can promote inclusion body formation for some proteins while growth at lower temperatures (e.g., 30°C, 25°C, 15°C) may not. (2) The lower temperature also decreases protease activity. Disadvantages are the following. (1) Growing the culture at a lower temperature will significantly slow the growth of *E. coli*, and so a longer induction period (e.g., overnight) may be necessary to obtain a sufficient amount of recombinant protein. (2) Growing the culture at a lower temperature will slow the rate of protein synthesis, possibly keeping recombinant proteins from saturating cellular folding machinery and aggregating [47]. (b) *Addition of cofactors*: potential cofactors should be added to the growth medium. Some proteins cannot properly fold without their cofactor and therefore can form inclusion bodies. (c) *pH alteration*: alteration of pH of growth medium can improve expression. pH is one culture variable that can affect proteolytic activity, secretion, and protein production levels [48]. 

### 5.12. Metabolic Load on the Organism

Regardless of the nature of the foreign gene or the design of the fermenter, the introduction of an exogenous plasmid into an *E. coli* cell is bound to impose some metabolic load [49].

#### 5.12.1. Solution

This may be avoided (1) by integrating the foreign gene into the *E. coli* chromosome through the use of a defective bacteriophage lambda lysogen carrying the foreign gene [50], (2) by the direct insertion of a foreign gene into a specific site on the host chromosome [51]. 

## 6. Conclusion 

While the efficient expression of foreign genes in *E. coli* is dependent on a number of factors, it is nevertheless reasonable to expect that most foreign genes may be expressed at high levels in *E. coli* and that this expression will be amenable to scale-up. Although the strategy of gene expression and scale-up is likely to vary, there are more similarities than differences from one gene to the next, resulting in the development of a “systems” approach to the cloning, expression, and scale-up of enzyme genes in *E. coli*. The eventual objective of producing a desired protein in an economical heterologous host is influenced by a variety of factors. However, maximizing production of heterologous proteins for commercial application is still an art. We have begun to understand factors influencing the eventual production. These factors, described in detail in this paper are varied and at times poorly understood. Largely the approach remains empirical. However, our collective experience will permit us to rationalize our approach in designing heterologous production of commercially important enzymes in a variety of expression systems. Subsequent to production, stabilization, and formulation of proteins will pose significant hurdles in utilizing the natural biological catalysts and other proteins for therapeutic and industrial purposes.

## Figures and Tables

**Table 1:** Some enzymes which are produced in bulk quantities and their industrial applications.

Enzymes	Industrial applications
Protease	(i) Inclusion in detergent preparations
	(ii) Cheese making
	(iii) Brewing/backing industries
	(iv) Meat/leather industries
	(v) Animal/human digestive aids.

Amylase	(i) Starch processing industry
	(ii) Fermentation industry

Cellulases/ hemicellulases	(i) Brewing industry
	(ii) Fruit juice production
	(iii) Animal feed industry

Pectinases	(i) Fruit juice processing industry

Lipases	(i) Dairy industry (ii) Vegetable oil industry
	(iii) Leather industry

Glucose isomerase	(i) Production of high-fructose syrups

Lactase	(i) Hydrolysis of milk lactose
	(ii) Digestive aid

Cyclodextrin glycosyltransferase	(i) Production of cyclodextrins for pharmaceuticals and other industries

Penicillin acylase	(i) Production of synthetic penicillin

Carbohydratases	(i) Baking, brewing, confectionary, and fruit industries

**Table 2:** Frequency of arginine codon usage for four different species.

Codon	*Escherichia coli*	*Bacillus* *subtilis *	*Saccharomyces* *cerevisiae *	*Homo sapiens*
CGU	38	18	14	8
CGC	40	21	6	19
CGA	6	10	7	11
CGG	10	16	4	22
AGA	4	26	48	20
AGG	2	9	21	20
